# Study on Strength Behavior of Organic Soil Stabilized with Fly Ash

**DOI:** 10.1155/2017/5786541

**Published:** 2017-09-11

**Authors:** Bayshakhi Deb Nath, Md. Keramat Ali Molla, Grytan Sarkar

**Affiliations:** Department of Civil Engineering, Khulna University of Engineering & Technology, Khulna 9203, Bangladesh

## Abstract

The aim of this study is to investigate the effect of fly ash on the consistency, compactness, acidic properties, and strength of organic soil. The presence of organic content in the soil has detrimental impacts on the physical and strength behavior of soil. To investigate the effectiveness of fly ash in the stabilization of organic soil, two types of fly ashes (Type I and Type II) at different percentages were used. It is found that fly ash significantly reduces the plasticity index of the organic soil, whereas the liquid and plastic limits increase. The dry density of the fly ash-soil mixture increases significantly, while the water requirement reduces due to the addition of fly ash. The increase of dry density compromises higher strength. The increase of *q*_*u*_ with the increase of fly ash content is mainly due to the pozzolanic reaction of fly ash, although the reduction in water content results from the addition of dry fly ash solid. Moreover, Type I fly ash contributes a higher value of* qu* compared to Type II fly ash. This is attributed to the characteristics of fly ash including CaO and CaO/SiO_2_ ratio.

## 1. Introduction

Soil is one of the most important and primary media for any construction work. The strength and durability of any structure depends on the strength properties of soil. It has been found from several studies that, due to the detrimental characteristics of organic soil, the shear strength and bearing capacity of this soil are very low, while the compressibility is very high. In recent years, subgrades of roadways are generally constructed by replacing the underneath organic soil with granular soil named “cut and replace” or preloading to improve the engineering properties of soil, which requires a huge investment cost and effort. This cost of using granular soil can be minimized by blending and mixing the existing soil with a cementing admixture, known as chemical stabilization process. The process may include the blending of soils to achieve a desired gradation or the mixing of commercially available additives which may alter the gradation or the mixing of commercially available additives that may alter the gradation, texture, or plasticity or act as a binder for the cementation of soil [1]. Cementing admixtures including cement, lime, rice husk ash, and fly ash are widely used and have attracted the attention of researchers because of their low cost and high pozzolanic action [2–7].

Fly ash is the combustion product of subbituminous coal in electric power plants and requires to be landfilled. However, many countries have promoted the reuse of these types of wastes in the interest of sustainable construction. Therefore, the use of fly ash as a binding admixture not only improves the engineering properties of soil but also reduces the use of energy and greenhouse gases. Fly ash disperses the soil cement clusters into smaller clusters, thereby increasing the reactive surface for hydration and pozzolanic reactions [8, 9]. Due to these pozzolanic characteristics, the shear strength and bearing capacity of the organic soil can be increased by stabilizing it with fly ash. Fly ash reduces the plasticity index and shrinkage limit, which has a potential impact on the engineering properties of fine-grained soil [10, 11]. However, the effectiveness of the stabilization depends on the organic content present in the soil, which is not taken into account in the case of inorganic soil [12–16].

Khulna is the third largest metropolitan and second port city in Bangladesh, which is located at the southwestern region of the country, bounded by the latitude from 22°46′0′′ to 22°58′0′′ north and longitude 89°28′0′′ to 89°37′0′′ east. It is a linear shaped city with an elevation from 9 m in the north to 2 m towards the southwest direction from the mean sea level, comprising very soft to soft consistency up to 20 ft. from the ground surface level. The generalized investigation results showed that the soils in the northern and southern part are mainly composed of clay with silt and organic deposits; the middle eastern part comprises sand with some clay and a moderate type of soil is found in the middle western part which includes mainly silt with clay and sand. It has been found that the organic soil exists here at a depth of 10 to 25 ft. below the existing ground surface and the organic content of this soil ranges from 5 to 70% and even more in some instances. It is seen that most of the region of this area contains fine-grained soil with some organic deposits which have lower bearing capacity and are not good for shallow foundations for buildings and roadways. However, Khulna City is progressing with several development projects including construction of high-rise buildings, oil storage tanks, long span bridges, harbors, port structures, flood protection embankments, and barrages. Since all structural loads are transferred to soil, the characteristics of soil play a vital role in the safety of these structures [17]. In this study, the effectiveness of fly ash on the strength and density of the organic soil of Khulna region was investigated. In addition, the quantity of fly ash that optimizes the unconfined compressive strength of stabilized organic soil was also estimated at different curing days.

## 2. Materials and Methods

### 2.1. Soils

The soft organic soil having organic content of 36.9% was collected from Beel Dakatia, Shiromoni, Khulna. Samples were collected within a depth of about 1.5 m from the existing ground surface. The collected soil was kept in a large polythene bag and dried in air for about 7 days.  Table 1 shows the physical and index properties of the organic soil ASTM D2974-14 [18]. It is seen that the soil contains high liquid limit, low unit weight, and high water content.

### 2.2. Fly Ashes

Fly ash is a very fine powdery material, composed mostly of silica which is a product of burning finely ground coal in a boiler to produce electricity. Two types of fly ash were collected from locally available cement industries in the southern part of Khulna. The chemical composition and physical composition of Type I and Type II are summarized in Table 2. According to ASTM C 618, Type I fly ashes classify as Class C ash and Type II fly ashes classify as Class F [19, 20]. It is found that Class C fly ashes are finer compared to Class F fly ashes, while the other physical properties remained the same. Moreover, the CaO and CaO/SiO_2_ content of Class F fly ashes is lower than that of the Class C fly ashes. Therefore, Class C fly ashes offer a more economical alternative as a soil stabilizing agent because of their pozzolanic characteristics. This type of fly ash provides the opportunity for applications where other activators would not be required [21].

### 2.3. Experimental Method

Standard Proctor compaction tests were carried out to determine the optimum moisture content and maximum dry density of all fly ash-soil mixtures according to ASTM D698-12e2 [22]. Cylindrical samples having a diameter of 38 mm and height of 76 mm, used in the UCS test, were prepared at their corresponding optimum moisture content and maximum dry density by static compaction. For curing, the samples were closely wrapped in a polythene bag and placed above water in a desiccator kept in a room. The unconfined compressive strength of the samples was assessed according to ASTM D5102-09 [23]. The index properties of the organic soil and fly ash treated soil were determined according to ASTM D2976-15 [24].

## 3. Results and Discussions

### 3.1. Index Properties

**Figure 1:**
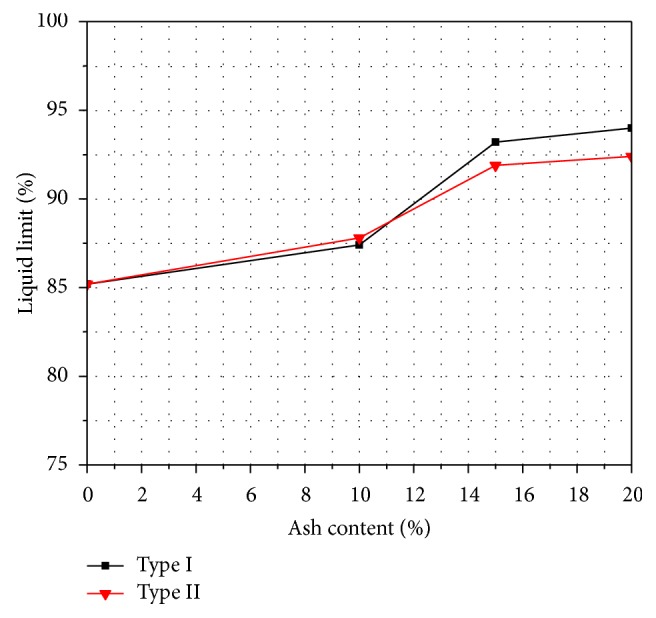
Variation of liquid limit with fly ash content.

**Figure 2:**
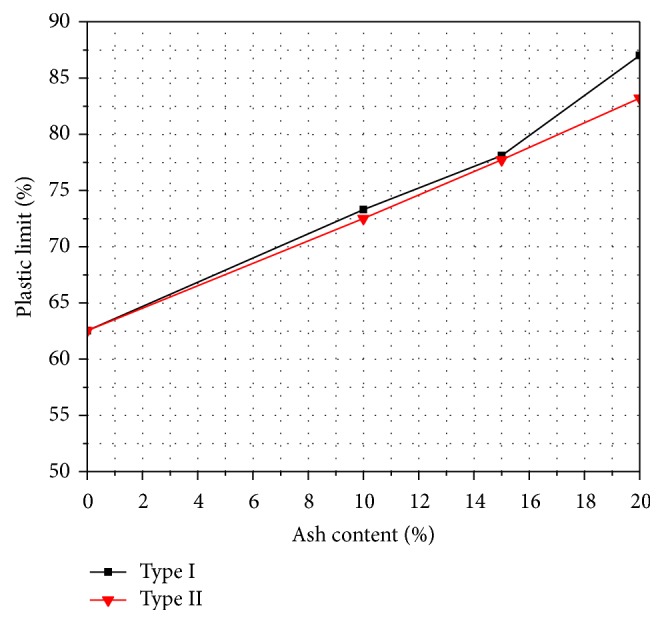
Variation of plastic limit with fly ash content.

Atterberg limit is very important for the characterization of soil within a broad category. The variations of liquid limit and plastic limit with varying percentages of fly ash are shown in Figures 1 and 2. It is seen that both the liquid limit and the plastic limit increase with the increase of both types of fly ash content. For example, for the Type I fly ash, the liquid limit ranged from 85 to 94% and the plastic limit ranged from 62 to 87%, thus resulting in a decrease of plasticity index values ranging from 22 to 7%, while in the case of Type II fly ash, the liquid limit ranged from 85 to 92%, plastic limit ranged from 62 to 83%, and plasticity index ranged from 22 to 9. Tastan et al. [25] showed similar results and explained that these beneficial changes in engineering properties are mainly attributed to cation exchange, flocculation of the clay, agglomeration, and pozzolanic reactions. For example, rapid and immediate changes in plasticity occurred due to the cation exchange and flocculation of clay.

**Figure 3:**
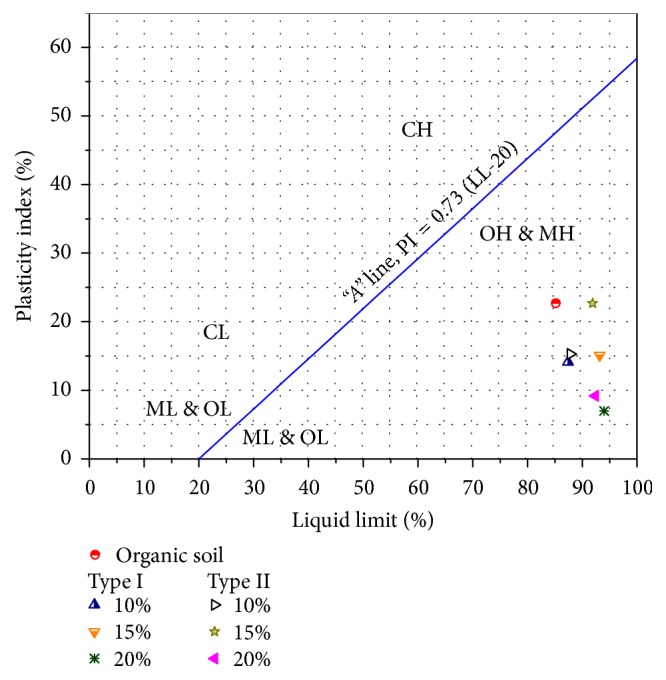
Plasticity chart showing the original and fly ash treated soil.

The plasticity chart shown in Figure 3 depicts the change in form of soil grains with the addition of fly ash. This curve represents the notion that the soil initially contained high plastic organic content and after the addition of ash it changed its plasticity. This change in form is attributed to the agglomeration of soil particles due to the production of calcium silicate gel. This gel coats the soil clasts, binding them together and filling the pores, resulting in a reduction of water absorption and shrinkage as described by Okagbue [26].

### 3.2. Compaction Characteristics

**Figure 4:**
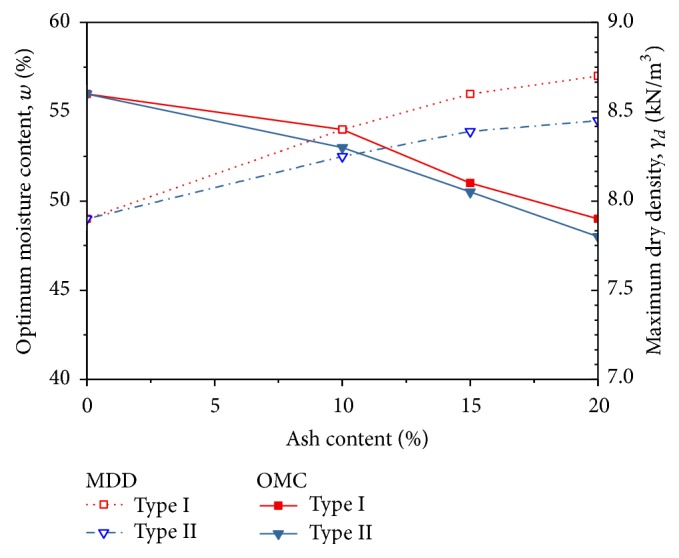
Variation of maximum dry density and optimum moisture content with ash content.

Figure 4 shows the effect of fly ash on the optimum moisture content (OMC) and maximum dry density of soil. It can be seen that the maximum dry density decreases with the increasing amount of fly ash, while the optimum moisture content gradually increases for both types of soil. This trend is similar to that found by Kaniraj and Havanagi [27]; they described that the decrease in the maximum dry density is attributed to the agglomeration and flocculation of clay particles through cation exchange reaction, leading to the occupation of a larger space as well as reducing the weight : volume ratio.

On the other hand, the optimum moisture content of soil increases with the increase of fly ash content as more water is required for the formation of the lime-like product, Ca(OH)_2_, and dissolution of this product into Ca^2+^ and OH^−^ ions, in order to supply more Ca^2+^ ions for the cation exchange reaction. Besides that, the finer the surface area, the more the water required to provide good lubrication. The ash content also decreases the quantity of free silt and clay fraction, forming coarser materials, which occupy larger spaces for retaining water.

### 3.3. pH

**Figure 5:**
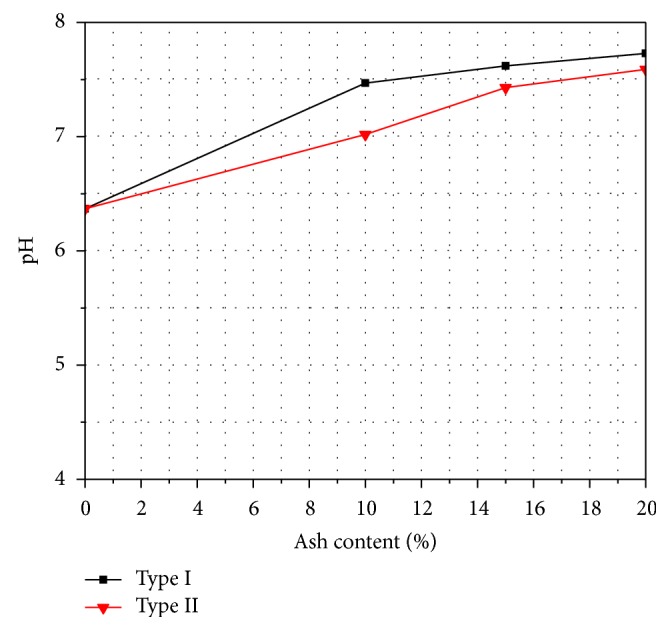
Variation of UCS with ash content.

Kitazume [28] showed that cement and blast furnace slag help to gain strength significantly, when organic content and humic acid are less than 15% and 0.9%, respectively, or pH is higher than 5, while humic acid consumes the calcium ions in the binder of organic soil and does not always indicate significantly high strengths. Tastan et al. [25] also reported that there is no apparent relationship between *qu* or resilient modulus and mixture pH. The pH of the organic soil and ash treated soil was determined according to ASTM D2976-15 [24].  Figure 5 shows the variation of pH with ash content and it is found that the pH value is higher for Type I fly ash treated soil compared to Type II ash treated soil.

### 3.4. Unconfined Compressive Strength (UCS)

**Figure 6:**
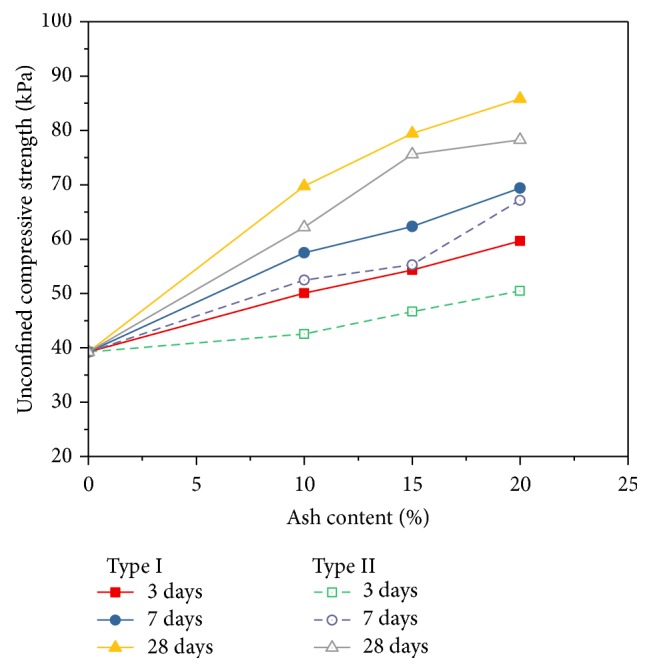
Variation of UCS with ash content.

Unconfined compressive strengths (*q*_*u*_) of the soil–fly ash mixtures prepared at their respective moisture content are shown as a function of fly ash type in Figure 6. Triplicate specimens were tested for unconfined compressive strength as quality control, and the averages of these tests are reported as results. Addition of fly ash to the organic soils resulted in a significant increase in *q*_*u*_ relative to that of the unstabilized soil. It is clear from the figure that the final *q*_*u*_ achieved varies depending on the organic soil and the fly ash. This is in contrast to the findings reported for inorganic soils stabilized with different fly ashes by Edil et al. [29], for which final strengths were comparable, although strength factors varied.

The illustration shows that there is a rapid increase of UCS with the addition of ash content up to 15%, and for the further 5% ash content, the UCS value increased gradually. The reason for this improvement is the formation of cementing gels (hydrate) due to the reactions between CaO of ash with Al_2_O_3_ and SiO_2_ of soil. This results in the agglomeration of large size particles and causes the increase in compressive strength. In addition, the compressive strength of fly ash treated soil increases with the increases of curing times. It is also seen that compressive strength of Type II fly ash treated soil is higher compared to Type I fly ash treated soil.

## 4. Conclusions

The effectiveness of fly ash for the improvement of unconfined compressive strength was investigated. Two types of fly ash, Type I (classified as Class C) and Type II (classified as Class F), were used to stabilize the organic soil at Khulna City, Bangladesh. Based on the test results, the following conclusions can be drawn:

- The moisture content decreases and the dry density increases gradually for the addition of both types of fly ash.
- Both values of liquid limit and plastic limit increase and the plasticity index decreases with increasing percentages of both types of fly ash content.
- The unconfined compressive strength increases with the increasing percentages of fly ash content for both types of fly ash.
- The unconfined compressive strength also increases with the increase of curing period.
- The pH value increases with the increasing percentages of fly ash content for both types of fly ash stabilized organic soil.
- The Type I fly ash stabilized organic soil can produce higher strength and dry density than the Type II fly ash stabilized organic soil. So, Type I fly ash is preferable over Type II fly ash in order to improve the quality of soil.

Finally, it can be said that the properties of organic soil can be improved by using fly ash but the amount of this improvement depends on the characteristics of organic soil as well as the properties and the amount of fly ash. Further study is required to obtain the optimum amount of fly ash.

## Figures and Tables

**Table 1:** Index properties of soil.

Property name	Values
Unit weight (kN/m^3^)	11.17
Liquid limit, *w*_*L*_ (%)	85.2
Plastic limit, *w*_*P*_ (%)	62.53
Plasticity index, *I*_*p*_ (%)	22.67
Shrinkage limit, *w*_*s*_ (%)	28.19
Shrinkage ratio, SR	1.24
Water content, *w* (%)	87.12
Specific gravity, *G*_*s*_	2.19
pH	6.37
Organic content (%)	36.9
Classification	
USCS	OH
AASHTO	

**Table 2:** Chemical composition and physical properties of fly ash.

Composition or property	Type I	Type II
Chemical composition		
Silica (SiO_2_)	50.2	65.6
Alumina (Al_2_O_3_)	22.5	25
Iron oxide (Fe_2_O_3_)	5.17	6
Lime (CaO)	20.3	1.78
Sulphates (SO_3_)	0.39	0.3
Magnesia (MgO)	0.51	0.7
Physical properties		
Fineness (specific surface) by Blaine's	374.6	300
permeability method (m^2^/kg)		
Sieving on 45-micron residue (%)	17.82	7.05
Loss on ignition (%)	4.02	4.00
Insoluble residue (%)	90.74	96.00
Moisture (%)	0.18	2
